# Editorial Note: *In silico* knockout studies of xenophagic capturing of *Salmonella*

**DOI:** 10.1371/journal.pcbi.1012998

**Published:** 2025-04-09

**Authors:** The PLOS Computational Biology Editors

After publication of this article [1], concerns were raised about the replicability of the results based on the descriptions provided in [1] and the publicly available software.

The corresponding author stated that the previous, binary format (S1 and S2 Files published with [1]) for the Petri net models produced by the MonaLisa software [2] has been changed to SBML format since the publication of [1], and the current version of the MonaLisa software [2] no longer supports the binary ‘.mlproject’ format. They provided SBML layout files (S1 and S2 Files here) to conserve the layout given the layout of the previous Petri net models (S1 and S2 Files published with [1]) can no longer be viewed, as well as the *Salmonella* model and the invariants’ results (S3 and S4 Files). The corresponding author stated that the underlying graph structure is not affected, and only the layout has changed. They also noted that the software used to produce the *in silico* knockout matrix in Fig 5 in [1] can be found at https://sourceforge.net/projects/molbi-isiknock/files/, including the Java source code.

A member of the *PLOS Computational Biology* Board verified that the MonaLisa program [2] could be run with the MonaLisa.jar file available on GitHub [3] to reproduce the results reported in [1]; however, they noted that the analyses appear to run correctly in Windows only.

The corresponding author stated that using Linux Fedora on Lenovo and Unix workstations, the *Salmonella* model gives the same figure and transition invariants as using Windows, but the place-bordered model does not as recent C++ versions for Linux are not backwards compatible.

The *PLOS Computational Biology* Editors issue this Editorial Note to give readers the above information and the conditions for replicating the results in [1].

## Supporting information

S1 File Edited SBML file and analysis results.
(XML)

S2 File Edited SBML file and analysis results.
(XML)

S3 File The invariants’ results.
(INV)

S4 File The *Salmonella* model results.
(INV)
